# Bacterial Peroxidase on Electrochemically Reduced Graphene Oxide for Highly Sensitive H_2_O_2_ Detection

**DOI:** 10.1002/cbic.202200346

**Published:** 2022-07-20

**Authors:** Sheetal K. Bhardwaj, Tanja Knaus, Amanda Garcia, Ning Yan, Francesco G. Mutti

**Affiliations:** ^1^ Van't Hoff Institute for Molecular Sciences HIMS-Biocat & HetCat University of Amsterdam Science Park 904 1098 XH Amsterdam The Netherlands

**Keywords:** bacterial peroxidases, biocatalysis, biosensors, DyP peroxidases, graphene oxide electrodes, H_2_O_2_ detection

## Abstract

Peroxidase enzymes enable the construction of electrochemical sensors for highly sensitive and selective quantitative detection of various molecules, pathogens and diseases. Herein, we describe the immobilization of a peroxidase from *Bacillus s*. (BsDyP) on electrochemically reduced graphene oxide (ERGO) deposited on indium tin oxide (ITO) and polyethylene terephthalate (PET) layers. XRD, SEM, AFM, FT‐IR and Raman characterization of the sensor confirmed its structural integrity and a higher enzyme surface occupancy. The BsDyP‐ERGO/ITO/PET electrode performed better than other horseradish peroxidase‐based electrodes, as evinced by an improved electrochemical response in the nanomolar range (linearity 0.05–280 μM of H_2_O_2_, LOD 32 nM). The bioelectrode was mechanically robust, active in the 3.5–6 pH range and exhibited no loss of activity upon storage for 8 weeks at 4 °C.

## Graphical abstract

**A bacterial peroxidase (BsDyP)** from the “dye‐decolorizing” family was immobilized on electrochemically reduced graphene oxide (ERGO) deposited on indium tin oxide (ITO) and polyethylene terephthalate (PET) layers to create a mechanically robust biosensor with a shelf‐life of eight weeks. This biosensor was characterized for the highly selective and sensitive detection of hydrogen peroxide (linearity 0.05–280 μM, LOD 32 nM).

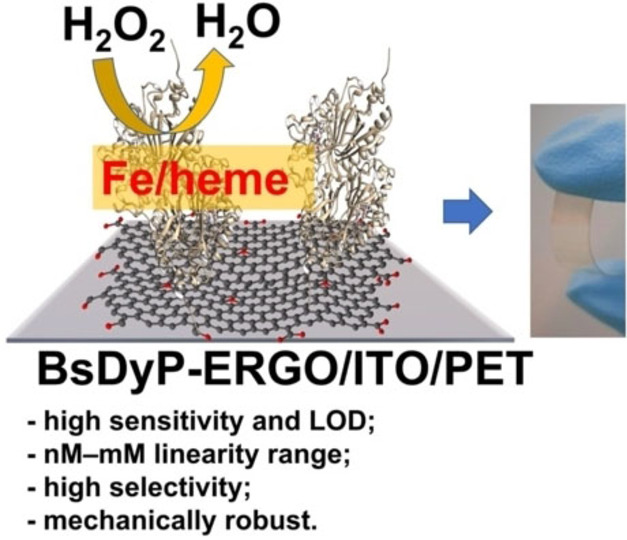

## Introduction

Enzyme‐based electrochemical sensors have the advantage of possessing high selectivity for detection in a complex environment and therefore find applications in many areas, including non‐invasive disease diagnosis, drug screening, food quality control and environmental monitoring.^[1]^ In this context, the accurate, rapid and online monitoring of H_2_O_2_ concentrations is of particular interest because it is a cellular metabolite whose production is connected to diseases such as cancer, diabetes, and cardiovascular and neurodegenerative disorders.^[2]^ Furthermore, H_2_O_2_ is a (by)product of multiple reactions that are relevant for biosensing in the food, medical, chemical and environmental sectors.[^1e^, ^3^] Available methodologies to detect H_**2**_O_**2**_ include the use of spectrometry, photometry, fluorimetry, titrimetry and chemiluminescence.^[4]^ Although these techniques offer high accuracy, they are often time‐consuming and cost‐ineffective, thereby undermining their suitability for high throughput analysis. Enzyme‐based electrochemical sensors can overcome these limitations by enabling simple, economically viable, high‐sensitive and real‐time monitoring of H_2_O_2_.[^3a^, ^5^] However, the construction of a high‐performance sensing device requires a combination of enzymes and electrode material with the optimal physicochemical properties and compatible methods to achieve stable enzyme immobilization.[^1b^, ^1c^, ^1e^, ^6^]

Among the category of heme‐dependent peroxidases (EC 1.11.1.X), horseradish peroxidase (HRP) and soybean peroxidase have been the preferred choices for H_2_O_2_ sensing.[^3^, ^5^, ^7^] These peroxidases are often used in combination with other H_2_O_2_‐producing enzymes for the detection of various metabolites (e. g., glucose, biogenic amines, glutamate, choline, uric acid, cholesterol, lactose) in biosensing applications.[^1b^, ^3a^, ^8^] Furthermore, they are used as an antibody labeling in enzyme‐linked immunosorbent assay (ELISA) tests for the detection of toxins, cancers, pathogens, etc.[^8a^, ^9^] However, plant‐derived peroxidases such as HRP are not ideal candidates for sensing applications because their challenging recombinant production affords low yields of active enzyme.[^5^, ^10^] Therefore, these enzymes are still produced by extraction from plant roots and commercialized as crude preparations containing impurities that can interfere with the analyte by giving false signals or reducing selectivity, and/or be toxic and preclude *in vivo* biosensing.^[10]^ Conversely, bacterial peroxidases from the “dye‐decolorizing” family (DyP, E.C.1.11.1.19) are attracting increased interest in chemical and biotechnological applications because they can be efficiently overexpressed in *E. coli* in high yield. They also exhibit high stability and can be secreted from the bacterial cell through the native TAT (i. e., transactivator of transcription) secretion system.^[11]^ The latter feature can be valuable for cost effective enzyme production in industrial settings due to the facilitated isolation procedure. As a novel family of enzymes for biotechnological applications, only a few works on DyP peroxidases have been published for biosensing. For instance, the electro‐catalytic properties of DyP from *Pseudomonas putida* MET94 (PpDyP) was explored.^[12]^ This enzyme and variants thereof were immobilized onto SAM/Ag for H_2_O_2_ detection. This biosensor exhibited a dynamic range of 1–200 μM and a sensitivity of 1.4 A M^−1^ cm^−1^ for the detection of H_2_O_2_.^[13]^

Various nanomaterial‐based electrodes including Au nanoparticles,[^7c^, ^7d^] carbon nanotubes,[^7e^, ^14^] semiconductors,^[15]^ graphene and derivatives thereof[^3b^, ^16^] can enable electron‐transfer from the immobilized enzyme to the electrode. However, many of those do not offer abundant anchoring sites due to the small surface area and rely on non‐covalent immobilization. Therefore, it remains a challenge to construct highly sensitive and stable peroxidase‐based electrochemical sensors.

Herein, we present a flexible, transparent and long‐term stable biosensor for the detection of H_2_O_2_ as low as to 32 nM. The biosensor consists of a layer of polyethylene terephthalate (PET) that is coated with indium tin oxide (ITO), onto which electrochemically reduced graphene oxide (ERGO) is deposited. A DyP peroxidase from *Bacillus subtilis* KCTC2023 (BsDyP),^[11c]^ which is recombinantly produced in *E. coli* with an additional N‐terminal poly‐histidine tag for simple and fast purification, is ultimately linked to the ERGO layer through the generation of covalent amide bonds. These bonds guarantee the robustness of immobilization with high surface coverage and stability during biosensor operation. Important features of the novel sensing device are high levels of sensitivity and selectivity, enhanced stability, and biocompatibility along with the possibility to use highly pure and active bacterial peroxidases.

## Results and Discussion

### Physicochemical and structural characterizations of GO, ERGO/ITO and BsDyP‐ERGO/ITO assembly

**Figure 1:**
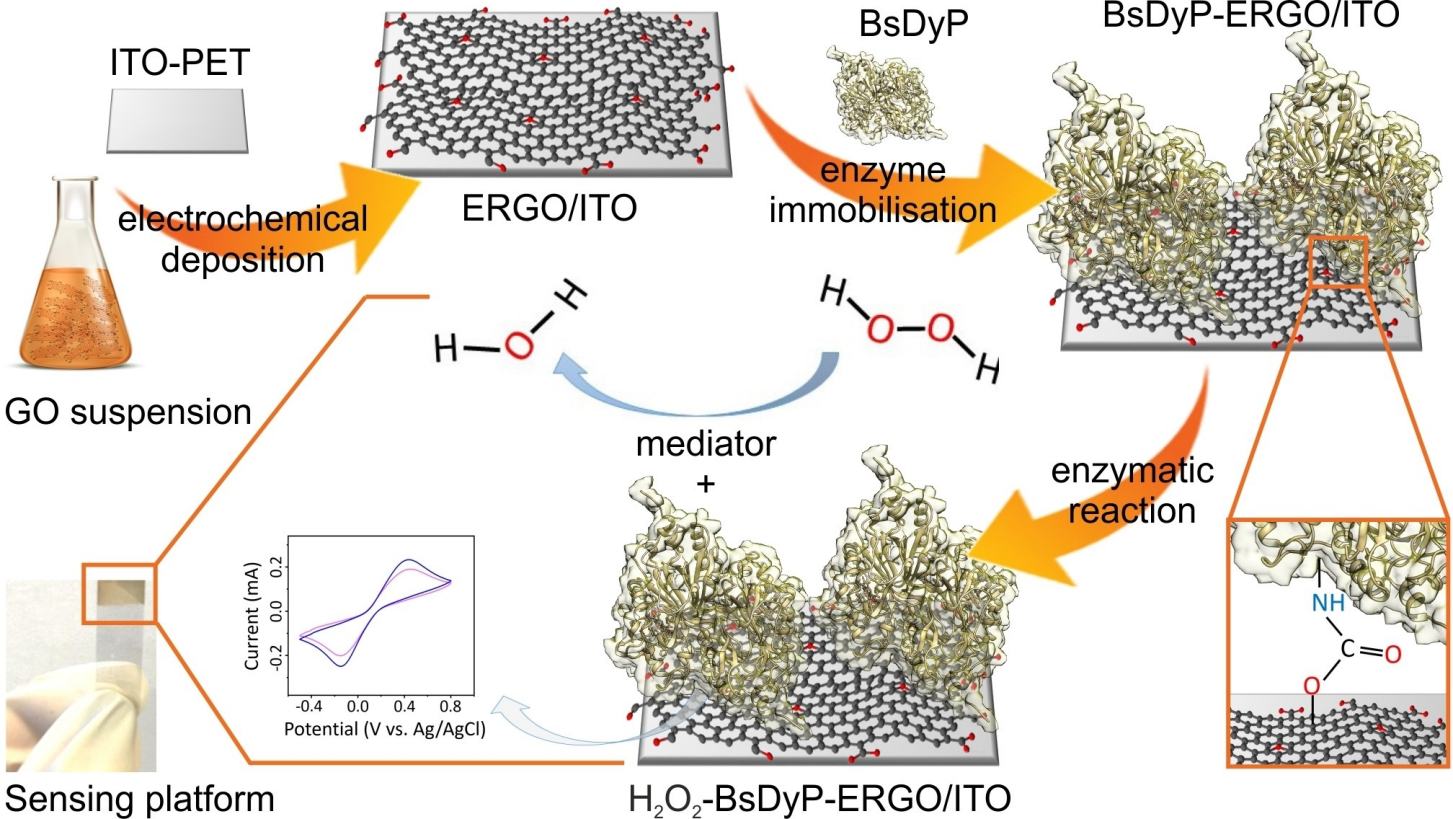
Schematic workflow for the preparation of the BsDyP‐ERGO/ITO/PET electrochemical biosensor for the quantitative detection of H_2_O_2_. Detailed depiction of biochemical reactions is reported in Scheme 1.

Figure 1 depicts a schematic workflow for the preparation of the BsDyP‐ERGO/ITO/PET electrochemical biosensor. In general, we used ITO/PET in this work except for FT‐IR, Raman and XRD characterization for which ITO/glass is needed to avoid the noise of PET peaks in the ERGO/ITO samples. Therefore, the acronym ITO is herein referred to ITO/PET or ITO/glass.

The graphene oxide (GO) was prepared using the modified Hummers’ method (see Supporting Information section 1.4 for details).[^16g^, ^17^] Next, GO was deposited and reduced on the ITO electrode in the manner reported in previous works.[^16d^, ^16g^] This electrophoretic deposition was modified to enable the preferential deposition of GO possessing more defective domains, which are ideal anchoring sites for large biomolecules such as enzymes (Figure 1, *vide infra*). XRD characterization of graphite, GO and ERGO/ITO is reported in Supporting Information, Figure S6. Finally, BsDyP was grafted onto the surface of the resulting ERGO/ITO electrode by reacting the carboxylic acid moieties of the ERGO layer with the amine moieties of the side chains of the enzyme's amino acid residues (e. g., l‐lysine residues) through EDC chemistry. The resulting assembly is the BsDyP‐ERGO/ITO/PET biosensor.

**Scheme 1:**
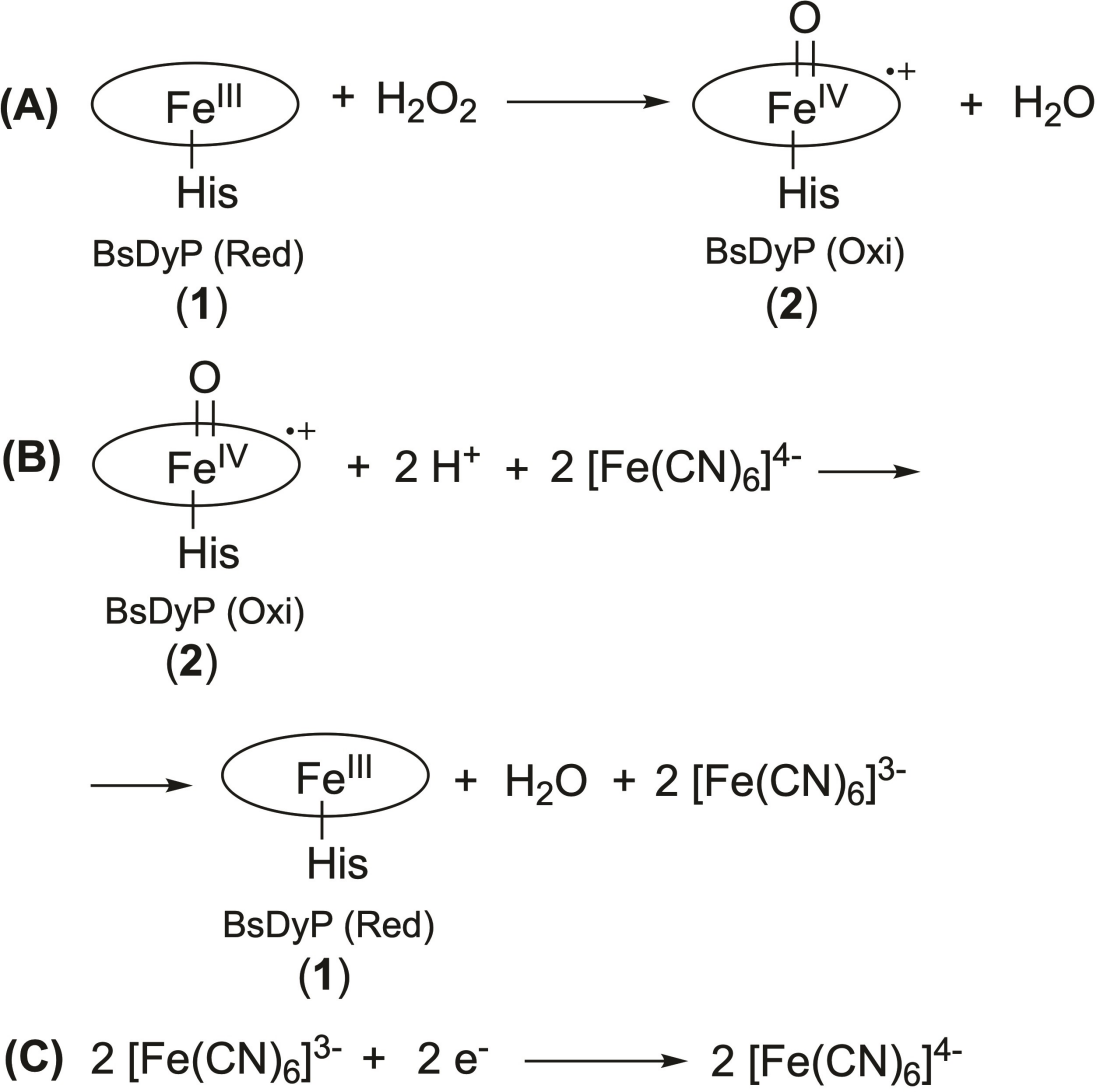
Biochemical reactions at the BsDyP‐ERGO/ITO electrode. For details on the three‐electrode system, see Figure S3.

The catalytic part of the electrode is the heme‐dependent BsDyP that catalyzes the reduction of hydrogen peroxide to water.[^11a^, ^11c^] The biochemical reactions of the BsDyP‐ERGO/ITO electrode are shown in equations 1–3 (Scheme 1). The couple [Fe(CN)_6_]^4−^/[Fe(CN)_6_]^3−^ acts as a mediator between the enzyme's active site and the electrode surface to improve the electron transfer rate according to the mechanism.^[18]^

Step (A): the [Fe^III^‐heme] catalytic center of BsDyP (**1**) is oxidized to O=[Fe^IV^‐heme]^.+^ (**2**) via binding with H_2_O_2_ followed by heterolytic cleavage of the O−O bond. Therefore, this ferryl (tetravalent) ion weakly bonded to an oxygen atom comes from the reduction of H_2_O_2_. In this way, the two electrons necessary for the reduction of the enzyme are implicit on the species O=[Fe^IV^−His BsDyP]^.+^ (**2**).

Step (B): the species (**2**) reacts with two protons from the solution and two electrons from the mediator 2[Fe(CN)_6_]^4−^ to regenerate the resting state of the enzyme (**1**) and 2[Fe(CN)_6_]^3−^.

Step (C) describes the redox process of the mediator, which involves two electrons transfer.

Therefore, the BsDyP‐ERGO/ITO sensor is a mediator‐assisted sensing system for H_2_O_2_ detection.

**Figure 2:**
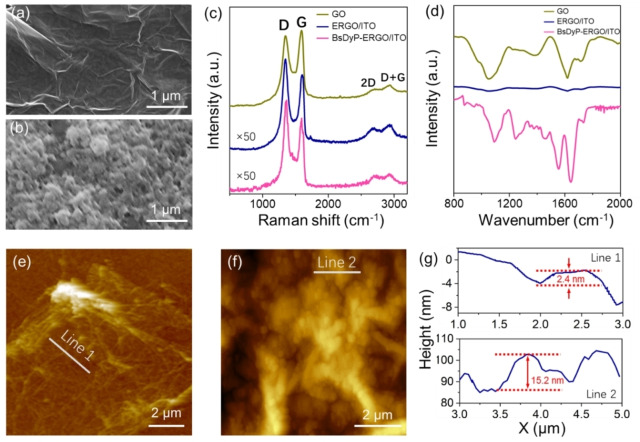
SEM images of (a) ERGO/ITO and (b) BsDyP‐ERGO/ITO electrode; (c) Raman spectra of GO, ERGO/ITO and BsDyP‐ERGO/ITO; (d) FTIR spectra of GO, ERGO/ITO and BsDyP‐ERGO/ITO; AFM images of (e) ERGO/ITO and (f) BsDyP‐ERGO/ITO electrode; the height profiles of the corresponding lines are shown in (g).

Figure 2a and b compare the scanning electron microscopy (SEM) images of ERGO/ITO and BsDyP‐ERGO/ITO. Figure 2a illustrates the uniformly distributed and transparent ERGO flakes that cover the entire ITO surface. These observed folded edges and wrinkles are the characteristic features of ERGO (see Supporting Information sections 1.4 and 1.5 for GO, ERGO synthesis and ERGO/ITO fabrication). After the immobilization of the BsDyP enzyme (see Supporting Information sections 1.6 and 1.7 for details), globular cluster‐like structures are formed on the surface of ERGO/ITO (Figure 2b). These clustered structures are the immobilized enzyme. Raman spectroscopy measurements were carried out to characterize GO, ERGO/ITO and BsDyP‐ERGO/ITO samples (Figure 2c). All spectra exhibited the characteristic D, G, 2D and D+G bands of the graphene derivatives (i. e., peak maxima at 1345, 1596, 2723, and 2938 cm^−1^, respectively), thus confirming the successful synthesis of the material.^[19]^ The D band is attributed to the presence of disorder, which might be both structural disorder, such as folding of graphene sheets, and the presence of residual oxygen. The G band represents the planar sp^2^ bonded carbon of the GO.

Notably, considering the relative intensity of the D and G bands in the samples, the deposited ERGO had a substantially greater I_D_/I_G_ ratio compared with that of GO (0.80 vs. 1.57),[^16e^, ^20^] suggesting a higher defect density on the ERGO sample. As the electrochemical reduction condition applied during the synthesis is unlikely to generate structural imperfections (e. g., defect domains),^[18b]^ we concluded that GO with more defects were selectively deposited on the ITO electrode, which might be associated with the distinct surface charge features of different GO in the colloid suspension. However, as the edge site of the defect domain contains abundant oxygen‐containing functional groups, this preferential deposition is beneficial for the anchoring of enzyme molecules. Finally, the Raman spectra of ERGO/ITO did not exhibit any significant change after the immobilization of BsDyP.

Next, we performed FT‐IR spectroscopy to confirm the correct immobilization of BsDyP onto ERGO/ITO (Figure 2d). The GO samples exhibited characteristic peaks at 1050, 1236, 1390, 1617, 1719, 3249, and 3357 cm^−1^, which correspond to the vibration modes of C−O (i. e., in alkoxy and hydroxyl groups), C=O (i. e., ketone moieties), C−O−C (i. e., epoxy group), OH and C−H bonds.^[21]^ As the coupled electrophoretic deposition and electrochemical reduction result in the formation of a thin film of ERGO on ITO, the signals become too weak to be analyzed for ERGO/ITO (Figure 2d, blue line). Conversely, a number of peaks re‐emerged upon the grafting of the enzyme to generate BsDyP‐ERGO/ITO. It is important to note the appearance of new peaks at 1637 cm^−1^ and in the range of 1205–1330 cm^−1^ that result from the formation of the new amide bonds between the BsDyP (i. e., amino groups) and the ERGO (i. e., carboxylic group). In fact, the peak at 1637 cm^−1^ and the peaks in the range of 1205–1330 cm^−1^ are due to N−H bending and C−N stretching of the amide functionality, respectively. Therefore, these observations confirm that the enzyme was anchored on the surface of ERGO via the amide covalent bonding. The two major bands of the protein IR spectrum, namely amide I and amide II, were also visible. Amide I band (ca. 1650 cm^−1^) is mainly associated with the C=O stretching vibration, whereas amide II band (ca. 1550 cm^−1^) is associated with N−H bending and C−N stretching vibrations.^[22]^

Additionally, atomic force microscopy (AFM) images (Figures 2e and f) clearly show the different modification process when BsDyP is immobilized on the electrode (Figure 2f). These images enabled us to obtain the average surface roughness (Ra) and root mean square roughness (Rq) of the fabricated surface (see Supporting Information, Table S2 for details), which well correlated with the SEM and FTIR results. The Ra and Rq values for the ERGO/ITO were 4.2 nm and 3.2 nm, respectively, with the height profile of 2.4 nm (see Figure 2g for the corresponding height profile of the selected line scans). After the immobilization of BsDyP onto the surface, the values of Ra and Rq increased to 90 nm and 60 nm, respectively, with the height profile of 15.2 nm. The drastic enhancement of surface roughness (from 4.2 nm to 90 nm for Ra and from 3.2 nm to 60 nm for Rq) following the immobilization confirms the presence of the biomolecules on the ERGO surface. Additionally, such roughness effect will influence the electrochemical response, as it will be discussed later on.

**Figure 3:**
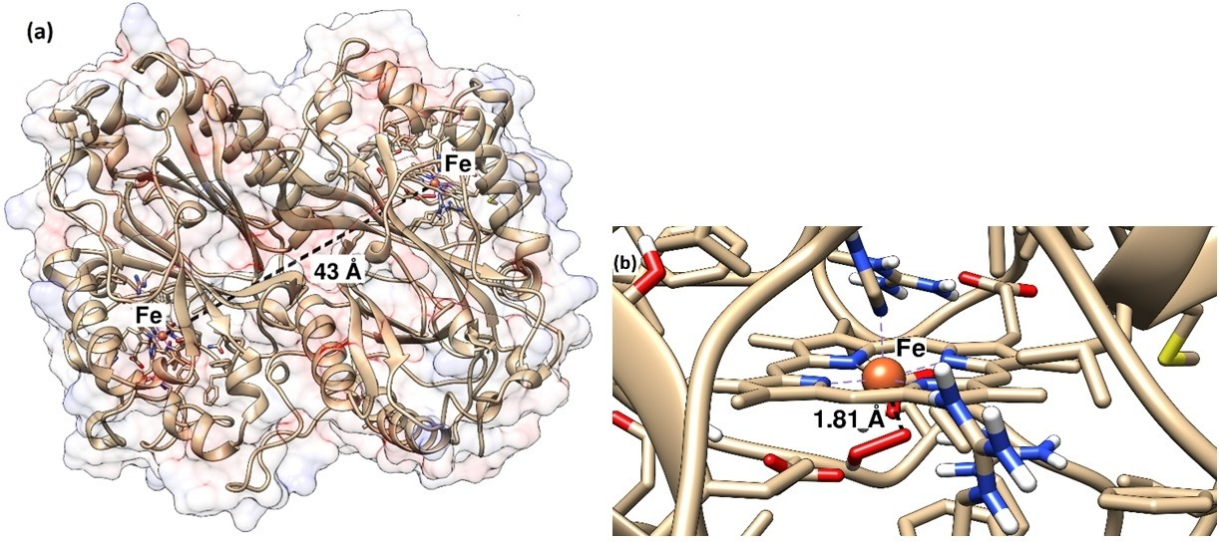
Homology model of the dimeric DyP from *Bacillus subtilis* (BsDyP): (a) overall enzyme structure with volume depicted in transparency and calculated as Coulombic electrostatic potential ‐ the distance between the Fe cations of the two heme groups is depicted with a black dash line; (b) closer view of one heme group (right from Figure 3a) showing the Fe cation coordinated to its HOO^−^ ligand.

From a structural enzymology perspective, the BsDyp enzyme used in our study exhibits a high degree of homology with another DyP from *B. subtilis* that was recently crystallized and biochemically characterized (PDB 6KMM and 6KMN).^[23]^ In particular, the structure of the crystallized BsDyP isoenzyme has 54 fewer amino acid residues at the N‐terminus than our BsDyP because this region was supposed to constitute a TAT secretion sequence (see Supporting Information section 2.1 for sequence alignment).^[23]^ However, the two BsDyP enzymes aligned for the remaining 363 amino acids residues and differed for only 12 of them (i. e., 96.7 % sequence identity in the overlapping region). Therefore, a homology model for our BsDyP was created using the structure of the BsDyP isoenzyme as a template (PDB 6KMN) along with four additional templates that were selected from the PDB database for their similarities with our target (see Supporting Information section 2.2, for details). Notably, all of the templates that exhibited the highest homology with our target BsDyP were crystallized as dimers. Accordingly, the homology model of our DyP was also obtained as a dimer with each monomer possessing a heme catalytic center (Figure 3). The dimer model of DyP shows that the two heme prosthetic groups ‐ one for each monomer ‐ are located at a distance ca. 43 Å from each other and are quite close to the protein surface (ca. 5 Å). The two iron centers are coordinated in the proximal position to either H_2_O_2_ (2.28 Å) or a HOO^−^ species (1.81 Å), respectively. A volume of 96.6×10^3^ Å^3^ and a surface area of 25.3×10^3^ Å^2^ were calculated using Coulombic electrostatic potential to describe the protein surface.

### Electrochemical characteristics of the electrode

A three‐electrode electrochemical cell configuration was used to characterize the BsDyP‐ERGO/ITO electrode. ITO/PET, Pt and Ag/AgCl were used as working, counter and reference electrodes (see Supporting Information, Figure S3 for details). Measurements were performed in 0.2 M acetate aqueous buffer electrolyte containing 5 mM of the redox couple [Fe(CN)_6_]^3−^/[Fe(CN)_6_]^4−^, which acts as the mediator. The reaction rates observed from this redox couple on the different electrodes give an idea about the surface chemistry and morphology.

**Figure 4:**
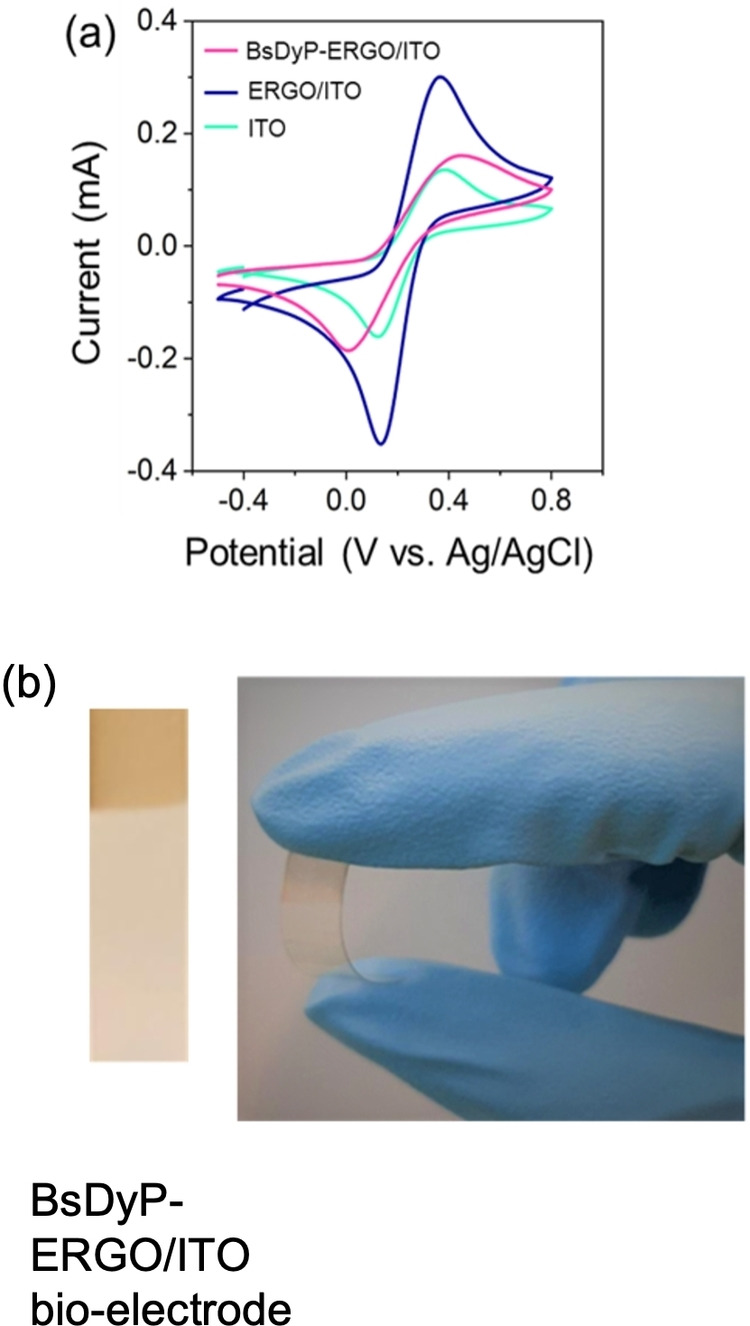
(a) Cyclic voltammograms (CV) of the ITO, ERGO/ITO and BsDyP‐ERGO/ITO electrodes recorded in 0.2 M acetate buffer (pH=4) containing 5 mM [Fe(CN)_6_]^3−/4−^ at scan rate (*ν*) of 100 mV s^−1^; (b) optical images of the flexible BsDyP‐ERGO/ITO electrode.

Figure 4a depicts the cyclic voltammograms (CV) of the ITO (turquoise line), ERGO/ITO (blue line) and the BsDyP‐ERGO/ITO (pink line) bio‐electrode in a potential range between −0.5–0.8 V. The anodic peak values (*I_pa_*) are recorded at 0.12, 0.31 and 0.16 mA for ITO, ERGO/ITO, and BsDyP‐ERGO/ITO, respectively. The increase in the magnitude of *I_pa_* for ERGO/ITO compared with ITO reflects the highly conductive nature and high surface area of ERGO film that is tightly bound to ITO surface. Therefore, the heterogeneous electron transfer ability of ERGO flakes possessing an extended π network enables efficient redox conversion.^[16a]^

As expected, the immobilization of BsDyP on the ERGO/ITO surface caused a decrease in *I_pa_* intensity from 0.31 to 0.16 mA due to the inherent insulating nature of proteins such as BsDyP, which commonly act as barriers in the electron transfer.^[18b]^ Nonetheless, the BsDyP‐ERGO/ITO electrode retained half of the conductivity of the ERGO/ITO carrier, which is remarkable given the size of the dimeric BsDyP enzyme. We attribute the retention of such a high level of conductivity in the final bio‐electrode to the position of the heme prosthetic group close to the surface of the enzyme scaffold. Additionally, the differences between the anodic and cathodic potentials for ITO, ERGO/ITO and BsDyP‐ERGO/ITO were always in the same range (∼0.24±0.08 V), and the calculated ratio of *I_a_*/*I_c_* was 0.85±0.2 (see Table S3 for details). As the *I_pa_*/*I_pc_* ratio is close to one, it confirms the electrode's quasi‐reversible nature.^[24]^

Notably, a high abundance of the active sites in the BsDyP‐ERGO/ITO electrode was achieved by optimization of the enzyme concentration on the electrode surface (ca. 5 nmol of enzyme monomer units on 0.25 cm^2^ of ERGO) as discussed in detail in Supporting Information sections 1.6–7.

### Characterization of BsDyP‐ERGO/ITO electrode for selective sensing of H_2_O_2_

**Figure 5:**
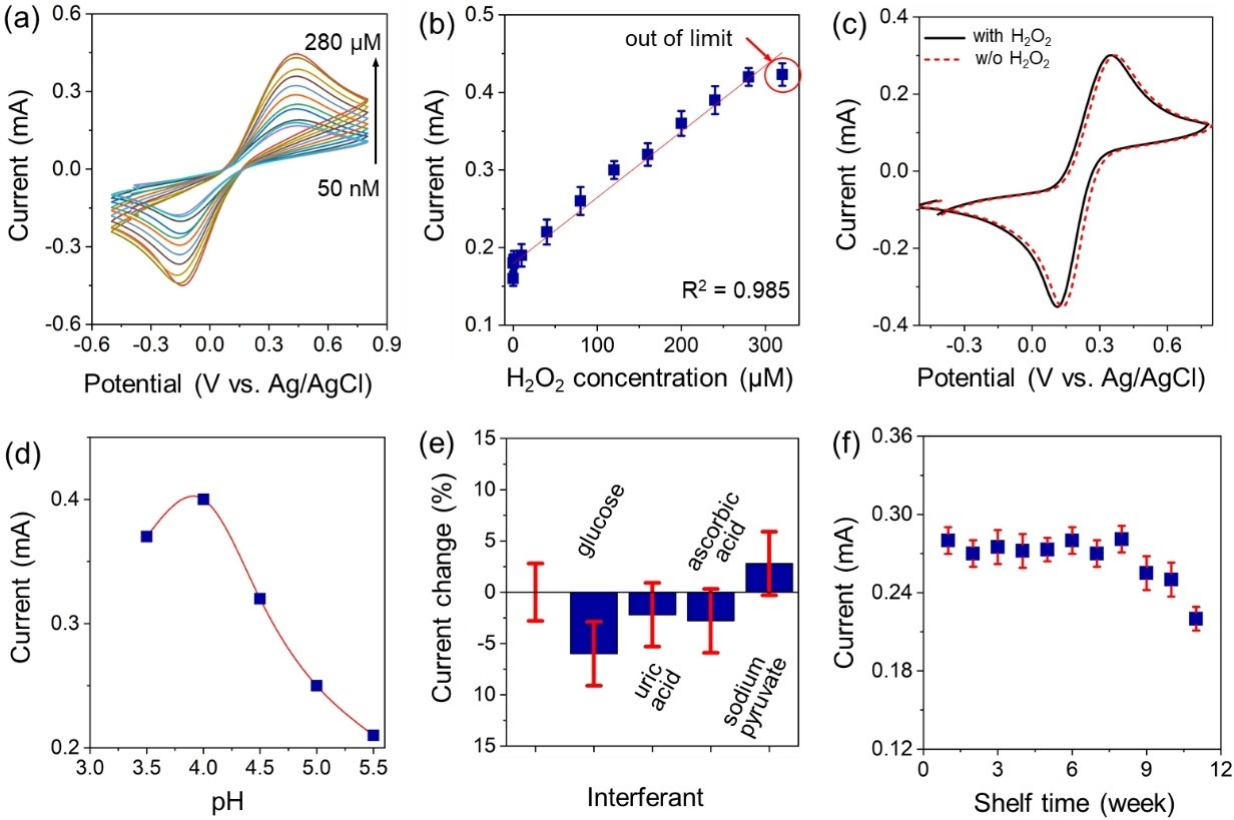
(a) CV response curves of BsDyP‐ERGO/ITO toward various H_2_O_2_ concentrations (0.05‐280 μM); (b) the plot of H_2_O_2_ concentrations versus *I_pa_*; (c) CV recorded for the ERGO/ITO with (150 μM) and without H_2_O_2_. (d) *I_pa_* recorded for the BsDyP‐ERGO/ITO at various pH. (e) Percentage change of *I_pa_* when BsDyP‐ERGO/ITO is exposed to the electrolyte containing glucose, uric acid, ascorbic acid and sodium pyruvate; (f) shelf‐life study for the BsDyP‐ERGO/ITO bioelectrode plotted by recording the *I_pa_* values in the CV measurements. All of the measurements were conducted in a 0.2 M acetate buffer (pH=4 if not stated otherwise) containing 5 mM of [Fe(CN)_6_]^3−/4−^; other than specified, the concentration of H_2_O_2_ and all the interferants was 200 μM.

We initially investigated the electrochemical response of BsDyP‐ERGO/ITO biosensor at H_2_O_2_ concentrations ranging from 0.05–320 μM. The CV scans were recorded in a 0.2 M acetate buffer (pH 4) containing 5 mM [Fe(CN)_6_]^3−/4−^ at a scan rate of 50 mV s^−1^ (Figure 5a). Figure 5b exhibits a linear correlation between the *I_pa_* and H_2_O_2_ concentrations up to 280 μM of H_2_O_2_. A deviation from linearity was observed above 280 μM of H_2_O_2_, thus signifying a lower response of the electrode above this value. These results demonstrate that the BsDyP‐ERGO/ITO biosensor is suitable for the detection of H_2_O_2_ in the range from 0.05–280 μM with a slope sensitivity of 8.8×10^−4^ mA μM^−1^ for 0.25 cm^2^ electrode surface (see Supporting Information section 3.4 for details). Additionally, the limit of detection (LOD) was calculated from the linear range of the fit using the “3*S_b_*/*m*” criteria wherein *m* is the slope of the linear range of the respective curve and *S_b_* is the standard deviation of the lowest concentration of the calibration curve; thus, the obtained LOD was 32 nM. Considering both the linearity range of response vs. concentration and the LOD value for H_2_O_2_, the performance of the BsDyp‐ERGO/ITO was superior to those of previously reported mediator‐assisted biosensors, which exhibited a narrower operative window and/or a significantly higher LOD value and/or a lower sensitivity.^[25]^ A comparison with other mediator‐assisted biosensors for the detection of H_2_O_2_ with HRP or catalase enzymes is reported in Supporting Information, Table S5. In comparison, our BsDyp‐ERGO/ITO electrode possesses a range of linearity that spans from the nanomolar to the hundreds of micromolar range, has a LOD as low as 32 nM and has elevated sensitivity. We have also conducted a control‐experiment using ERGO/ITO as electrode to ensure that the BsDyP‐ERGO/ITO biosensor can detect H_2_O_2_ due to the sensing capability originated from the enzyme (Figure 5c). As expected, using ERGO/ITO alone, we did not notice any *I_pa_* change when H_2_O_2_ (150 μM) was added to the electrolyte.

Additionally, the effect of the pH on the electrochemical response of BsDyP‐ERGO/ITO was evaluated in the pH range from 3.5–6 (Figure 5d) in different acetate buffer solutions containing [Fe(CN)_6_]^3−/4−^ and 200 μM of H_2_O_2_. The maximum magnitude of 0.39 mA for I_pa_ was observed at pH 4.0, which decreased to 0.38 mA at pH 3.5 and 0.35 mA at pH 4.5. A further decrease of the electrochemical response was detected at pH 5.0 (0.25 mA) and pH 5.5 (0.21 mA). These results reveal that the bio‐electrode operates at its highest response at pH 4.0; therefore, this pH value was selected for the continuation of the characterization. These data are in agreement with previous work on the biochemical characterization of BsDyP.^[11c]^ Interestingly, this biosensor is among the few reported in the literature that can operate in an acidic environment, thereby making it suitable for some industrial applications.

An important feature of an applicable biosensing device is its chemoselectivity for the analyte. Therefore, the chemoselective detection of H_2_O_2_ (200 μM) in 0.2 M acetate buffer supplemented with [Fe(CN)_6_]^3−/4−^ as electrolyte (5 mM) was assayed in the presence of various serum interferants such as glucose, ascorbic acid, sodium pyruvate and uric acid. Figure 5e depicts the percentage of current change (*I_pa_*) after adding the interferant into the buffer to yield a 1 : 1 molar ratio of H_2_O_2_ to interferant. As the current variations were in the range of 2–5 %, we concluded that the BsDyP‐ERGO/ITO bioelectrode can provide a selective and stable electrochemical response in the presence of different interferants that are common in biological samples. Although different molar ratios of H_2_O_2_ and interferant may influence the response of the biosensor, we deemed an in‐depth investigation as out of the immediate scope of this work.

In contrast, as additional important technical feature, we evaluated the shelf‐life of BsDyP‐ERGO/ITO within a ten‐week timeframe by measuring the electrochemical responses every seven days. The electrode was stored under refrigerated conditions (4 °C) between each measurement and the next. Figure 5f shows that significant loss of electrochemical activity was not detected after eight weeks; however, following this time, the performance gradually decreased to 90 % and 80 % of the initial value after nine and ten weeks, respectively. The exhibited shelf‐life of eight weeks of the BsDyP‐ERGO/ITO bioelectrode is remarkable; however, we trust that the life‐time of the bioelectrode can be further extended by more extensive optimization, in particular aimed at increasing the physicochemical stability of BsDyP by protein engineering.

## Conclusion

The combination of heme‐dependent peroxidases either with other H_2_O_2_‐producing enzymes or as a component in medical diagnostic tests enables the construction of electrochemical sensors for the quantitative, highly sensitive and selective detection of various molecules, pathogens and diseases. In this context, plant‐derived peroxidases are commonly applied, although they must be extracted from plant roots as non‐pure enzymes due to their difficult recombinant expression. In this work, we investigated a bacterial peroxidase DyP from *Bacillus subtilis*, which was produced in active form in *E. coli* and purified in high yield. BsDyP was efficiently immobilized through a covalent amide bond onto ERGO. Notably, we also described a facile approach of coating the defect‐rich ERGO on ITO to construct the electrode. The biosensor was characterized via XRD, SEM, AFM, Raman and FT‐IR spectroscopy, thus confirming the successful synthesis of the ERGO/ITO electrode and the immobilization of Bs‐DyP with elevated surface occupancy. The BsDyP‐ERGO/ITO exhibited a superior performance compared with other horseradish peroxidase‐based electrodes, namely a larger linearity range (0.05–280 μM) and/or a lower LOD value (32 nM) for the detection of H_2_O_2_. The bioelectrode also exhibited high mechanical flexibility, stability and chemoselectivity against a number of interferents as well as an excellent shelf storage. Currently, this bio‐electrode operates very efficiently at acidic pH and with a mediator. Therefore, it could be used in the food sector. Other studies in progress in our group are aimed at engineering the BsDyP to extend its pH tolerance and enable it to operate efficiently at pH above 6 and possibly without the need for a mediator. Thus, the engineered BsDyP‐based electrode would become suitable for medical diagnostic test both *in vitro* and *in vivo*. The simple and effective strategy of constructing a biosensing platform using affordable and biocompatible materials describe in this work can open new opportunities for rationally designing biosensors for various applications.

## Experimental Section

All experimental procedures are reported in the Supporting Information file. This file contains preparation of BsDyP‐ERGO/ITO biosensor, bioinformatic analysis and generation of homology model of BsDyP, characterization of ERGO/ITO and BsDyP‐ERGO/ITO.

## Conflict of interest

The authors declare no conflict of interest.

1

## Supporting information

As a service to our authors and readers, this journal provides supporting information supplied by the authors. Such materials are peer reviewed and may be re‐organized for online delivery, but are not copy‐edited or typeset. Technical support issues arising from supporting information (other than missing files) should be addressed to the authors.

Supporting Information

## Data Availability

The data that support the findings of this study are available in the supplementary material of this article.
