# Supplementary material for: Bacterial Peroxidase on Electrochemically Reduced Graphene Oxide for Highly Sensitive H2O2 Detection
Source: Chembiochem. 2022 Jul 20;23(17):e202200346. doi: 10.1002/cbic.202200346 (PMC9543142; doi:10.1002/cbic.202200346)
Supplement: Supplementary file 1 — Supporting Information [file CBIC-23-0-s001.pdf]

ChemBioChem

Supporting Information

Bacterial Peroxidase on Electrochemically Reduced Graphene Oxide for Highly Sensitive H_2O_2 Detection

Sheetal K. Bhardwaj, Tanja Knaus, Amanda Garcia, Ning Yan,* and Francesco G. Mutti*

1. Preparation of BsDyP-ERGO/ITO biosensor 2
1.1 Chemicals and equipment 2
1.2 Expression and purification of the peroxidase from *Bacillus subtilis* (BsDyP) and activity test 2
1.3 Preparation of stock solutions 3
1.4 Procedure for the synthesis of graphene oxide (GO) dispersion in water 3
1.5 Fabrication of electrochemically reduced graphene oxide (ERGO) film on ITO electrode 4
1.6 Immobilization of BsDyP onto ERGO/ITO electrode 4
1.7 Optimization for BsDyP concentration in fabricating BsDyP-ERGO/ITO bioelectrode 5
2. Bioinformatics analysis and generation of homology model of BsDyP 6
2.1 Amino acid sequences alignment between PDB 6KMN[3] and the BsDyP from this study (without poly-histidine purification tag) 6
2.2 Generation of homology model of BsDyP 6
3. Characterization of ERGO/ITO and BsDyP-ERGO/ITO 8
3.1 General information 8
3.2 XRD studies 8
3.3 Surface analysis 9
3.4 Electrochemical characterization 9
3.5 Calculation of the percentage of interference 10
4. References 11

1. Preparation of BsDyP-ERGO/ITO biosensor

1.1 Chemicals and equipment

Sodium nitrate ($NaNO_3$), graphite, hydrochloric acid (HCl), potassium permanganate ($KMnO_4$), hydrogen peroxide (H_2O_2, 30% v v^{-1}), ITO coated PET with the resistivity of ~ 60 Ω sq^{-1}, *N*-hydroxysuccinimide (NHS), *N*-(3-dimethylaminopropyl)-*N*-ethylcarbodiimide hydrochloride (EDC), 5-aminolevulinic acid, ferrous sulfate monohydrate ($FeSO_4 \bullet H_2O$) and 2,2'-azino-bis(3-ethylbenzothiazoline-6-sulfonic acid) diammonium salt (ABTS) were purchased from Sigma-Aldrich. Sulfuric acid (H_2SO_4, 98% w w^{-1}) was purchased from Acros Organics. Isopropyl-β-D-thio-galactopyranoside (IPTG) was purchased from Melford (UK). Pre-packed Ni-NTA HisTrap HP columns were purchased from GE Healthcare. Electrochemical analysis was conducted on a Gamry Potentiostat/Galvanostat Reference 600 Plus using a three-electrode system with ITO/PET as working electrode, platinum (Pt) wire as an auxiliary electrode and Ag/AgCl (saturated 3.5 M KCl) as a reference electrode in acetate buffer (pH 4.0, 0.2 M), containing 5 mM $[Fe(CN)_6]^{3-/4-}$ as a redox probe. The $[Fe(CN)_6]^{3-/4-}$ salts were added both in equimolar amount (2.5 mM each). In general, we used ITO/PET in this work except for FT-IR, Raman and XRD characterization for which ITO/glass is needed to avoid the noise of PET peaks in the ERGO/ITO samples.

1.2 Expression and purification of the peroxidase from Bacillus subtilis (BsDyP) and activity test

The synthetic gene encoding the BsDyP peroxidase bearing an additional poly-histidine tag was designed according to literature,[1] and cloned in *E. coli* BL21 DE3 cells. Enzyme expression and purification were performed as follows. For recombinant expression, 800 mL of LB medium supplemented with ampicillin (100 µg/mL) were inoculated with 15 mL of an *E. coli* overnight culture harboring the desired plasmid DNA. Cells were grown at 37 °C until an OD_{600} of 0.8-0.9 was reached and expression of protein was induced by the addition of IPTG (0.5 mM), 5-aminolevulinic acid (0.5 mM) and ferrous sulfate monohydrate (100 µM). Protein expression was carried out overnight at 20 °C. After harvesting of the cells (4 °C, 8 x 103 rpm, 10 min), the remaining cell pellet was re-suspended in lysis buffer (50 mM KH_2PO_4, 300 mM NaCl, 10 mM imidazole, pH 8.0) prior to cell disruption by ultrasonication. Protein purification was performed by Ni-NTA affinity chromatography using pre-packed Ni-NTA HisTrap HP columns previously equilibrated with lysis buffer. After loading of the filtered lysate, the column was washed with sufficient amounts of washing buffer (50 mM KH_2PO_4, 300 mM NaCl, 25 mM imidazole, pH 8.0); next, bound protein was recovered with elution buffer (50 mM KH_2PO_4, 300 mM NaCl, 200 mM imidazole, pH 8.0). After SDS-PAGE, fractions containing the desired proteins in a sufficient purity (> 90%) were pooled and dialyzed overnight against K_2HPO_4/KH_2PO_4 buffer (50 mM, pH 7.5) and then concentrated using Centripreps (Millipore). Purified BsDyP enzyme was aliquoted, shock-frozen in liquid nitrogen and stored at -80 °C. The final concentration of the protein was determined at 280 nm (ε_{280} = 42525 M^{-1} cm^{-1}). A typical protein yield of 41 mg L^{-1} of cell culture was obtained with an Rz value $(A_{soret,408}/A_{280})$ of 0.54 (approximately 38% of the protein was saturated with hemin; protein concentration: 427 µM, 20 mg mL^{-1} total protein).

Purified BsDyP was tested for its catalytic activity using 2,2'-azino-bis(3-ethylbenzothiazoline-6-sulfonic acid) diammonium salt (ABTS) as substrate. The assay reaction mixture (1 mL final volume in a 1.5 mL Eppendorf tube) consisted of sodium acetate buffer (200 mM, pH 3.65), ABTS (1 mM), H_2O_2 (100 µM) and BsDyp (3.4 µM). Additionally, negative controls that were devoid of (i) BsDyp or (ii) H_2O_2 were performed. After addition of the enzyme an immediate color change was observed indicating that BsDyp was purified as an active enzyme. Figure S1c shows the color change 20 sec after addition of BsDyP.

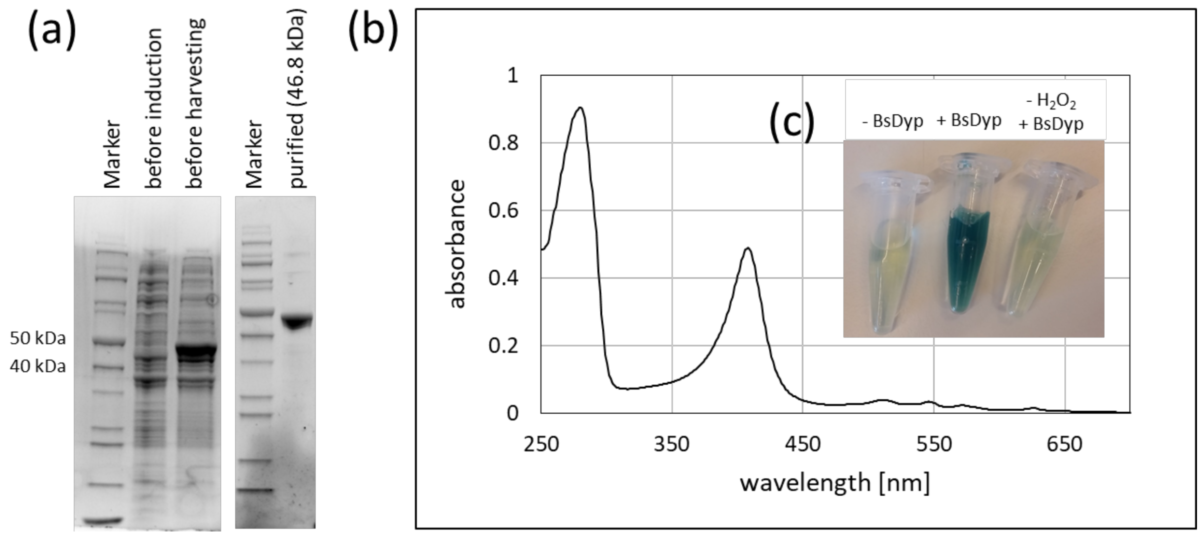

Fig. S1. (a) SDS-Pages for the expression of BsDyP and the purified enzyme. Marker: PageRuler™ Unstained Protein Ladder (ThermoFisher Scientific), (b) UV-vis absorbance spectrum of purified BsDyp (1:20 diluted) and (c) colorimetric activity test for BsDyp using ABTS as substrate (the image was taken 20 sec after addition of the enzyme to the assay mixture).

1.3 Preparation of stock solutions

Solutions of BsDyP (427 µM) was freshly diluted in PBS buffer (50 mM, pH 7.0) and henceforth named as solution A. Stock solution of H_2O_2 was prepared in water and stored at 4 °C. This stock solution was further diluted to prepare different concentrations of the analyte (H_2O_2). Solutions of EDC (0.4 M) and NHS were prepared freshly in deionized water.

1.4 Procedure for the synthesis of graphene oxide (GO) dispersion in water

Graphene oxide (GO) was prepared by modified Hummers' process.[2] Graphite powder was taken in a conical flask. $NaNO_3$ and H_2SO_4 were added in the flask already containing the graphite powder and stirred at 0 °C for 30 minutes (henceforth this solution is named as solution B). Next, $KMnO_4$ was added slowly to the solution B while stirring at room temperature. Solution was left for over-night stirring (henceforth, this solution is named as solution C). The color of the solution was green. Next day, H_2O was added dropwise to the solution C while stirring and the mixture was left for 30 minutes to maintain the temperature (henceforth, this solution is named as solution D). The color of solution changes from green to brown.

Later, when the temperature of solution reached room temperature, H_2O_2 (30% v v^{-1}) was added to the solution D while stirring and the color changed from brown to yellow. Thus, GO was prepared as shown in Figure S2. The prepared GO solution was washed with HCl (5% v v^{-1}) using centrifuge machine to remove the impurities. After washing with HCl, the solution was further washed with distilled water several times to change the pH from acidic to neutral. Finally, prepared GO water solution (1 mg mL^{-1}) was sonicated for 2 hours.

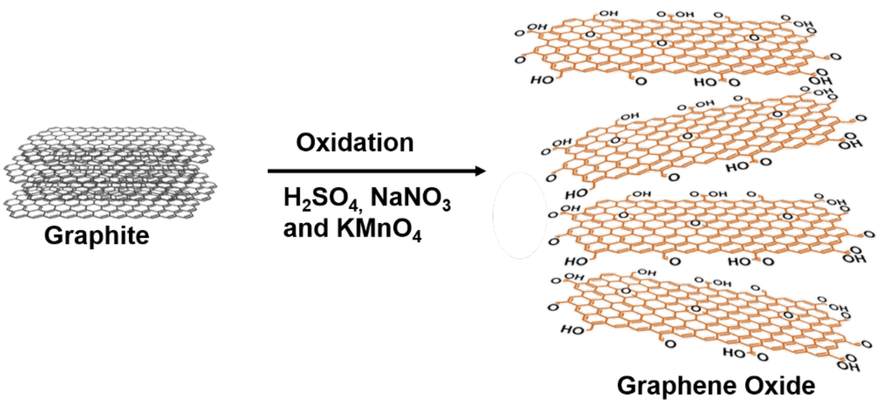

Fig. S2. Preparation of graphene oxide (GO) from raw graphite powder.

1.5 Fabrication of electrochemically reduced graphene oxide (ERGO) film on ITO electrode

The ITO/PET sheet was cut into electrodes of 1 cm x 2 cm dimensions. Electrodes were cleaned with acetone, ethanol, isopropyl alcohol and water. The ITO/PET electrodes were dried by N_2 blower. The above prepared GO suspension (1 mg mL^{-1}) was reduced on pre-cleaned ITO surface with application of a constant potential (-1.5 V) at neutral pH via chronoamperometric technique using a three-electrode system with ITO/PET as a working electrode (W), platinum (C) wire as an auxiliary electrode and Ag/AgCl as a reference electrode (R) as shown in Figure S3. Figure S3 shows the diagrammatic representation of three electrode system and camera captured image of the developed electrode.

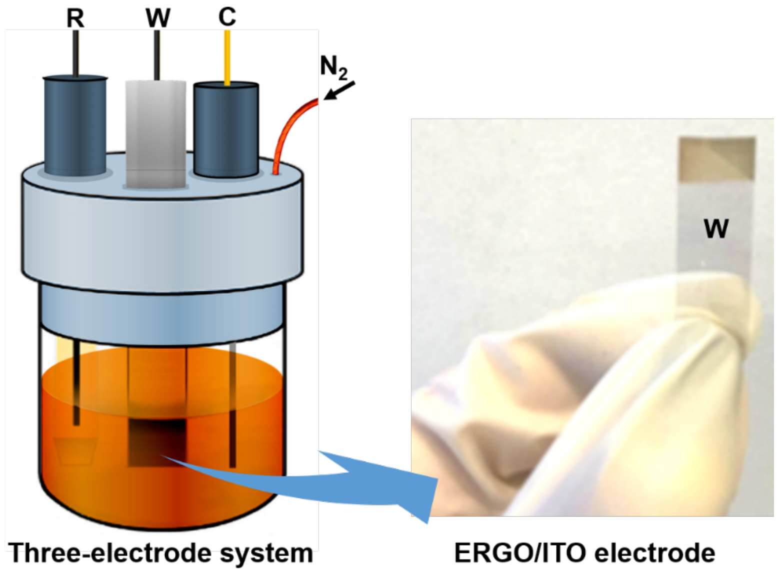

Fig. S3. Diagrammatic representation of three-electrode system (working electrode (W), platinum wire (C) as an auxiliary electrode and Ag/AgCl as a reference electrode (R)) and camera captured image of developed ERGO/ITO/PET electrode.

1.6 Immobilization of BsDyP onto ERGO/ITO electrode

BsDyP peroxidase was covalently attached to ERGO/ITO electrode via carbodiimide chemistry. For immobilization of the enzyme, 30 µL of solution A was mixed with EDC (0.4 M) and NHS (0.1 M). This enzyme mixed solution was added dropwise onto 0.25 cm^2 of ERGO/ITO electrode and kept in a humid chamber for 4 h. Next, the fabricated bioelectrode (BsDyP-ERGO/ITO) was washed with PBS solution (50 mM, 0.9% NaCl, pH 7.0) containing 0.05% Tween 20 to remove any unbound enzymes. The prepared BsDyP-ERGO/ITO bio-electrodes were refrigerated at 4 °C when not in use.

1.7 *Optimization for BsDyP concentration in fabricating BsDyP-ERGO/ITO bioelectrode*

The BsDyP-ERGO/ITO was optimized by pursuing measurements with four different concentrations of BsDyP stock solution in PBS buffer (i.e., 0.5 mg mL^{-1}, 1.0 mg mL^{-1}, 1.5 mg mL^{-1}, 2.0 mg mL^{-1}). The amount and concentration of BsDyP was optimized by drop-casting the different BsDyP solutions containing 0.4 M EDC and 0.1 M NHS over 0.25 cm^2 area of ERGO/ITO electrodes (named as electrodes B1, B2, B3 and B4 for 0.5 mg mL^{-1} solution, 1.0 mg mL^{-1} solution, 1.5 mg mL^{-1} solution and 2.0 mg mL^{-1} solution, respectively). After drop-casting the electrodes were placed in a humid chamber for 4 hours. The CV scans were recorded in acetate buffer (pH 4, 0.2 M) containing 5 mM $[Fe(CN)_6]^{3-/4-}$ at scan rate of 50 mVs^{-1}. The determined change in anodic peak currents (I_{pa}) of bio-electrodes B1, B2, B3 and B4 were plotted in a bar diagram (Fig. S4a). The maximum magnitude of peak current was observed for B2 (i.e., 1.0 mg mL^{-1}). Next, the B1, B2, B3 and B4 electrodes were tested for the detection of H_2O_2 (named as H1, H2, H3 and H4, respectively). CV scans were recorded at scan rate of 50 mVs^{-1} after dipping the electrodes B1, B2, B3 and B4 in a solution of H_2O_2 (100 µL of 5 µM stock solution) in acetate buffer (pH 4, 0.2 M) supplemented with 5 mM $[Fe(CN)_6]^{3-/4-}$. The value of the obtained I_{pa} were plotted in a bar diagram (Figure S3b). The observation of maximum change in electronic signals was observed for case H2. The large change in I_{pa} recorded for this BsDyP-H_2O_2 interaction suggested enhanced capability for detection of H_2O_2. Therefore, the optimum performance of the BsDyP-ERGO/ITO bioelectrode is obtained by using a 1 mg mL^{-1} concentration of the enzyme. On the one hand, for lower enzyme concentration, there is not enough enzymes to catalyze the optimal H_2O_2 reduction. On the other hand, when concentration is increased, the decrease in the anodic peak current is due to hindrance in charge transfer at the anode.

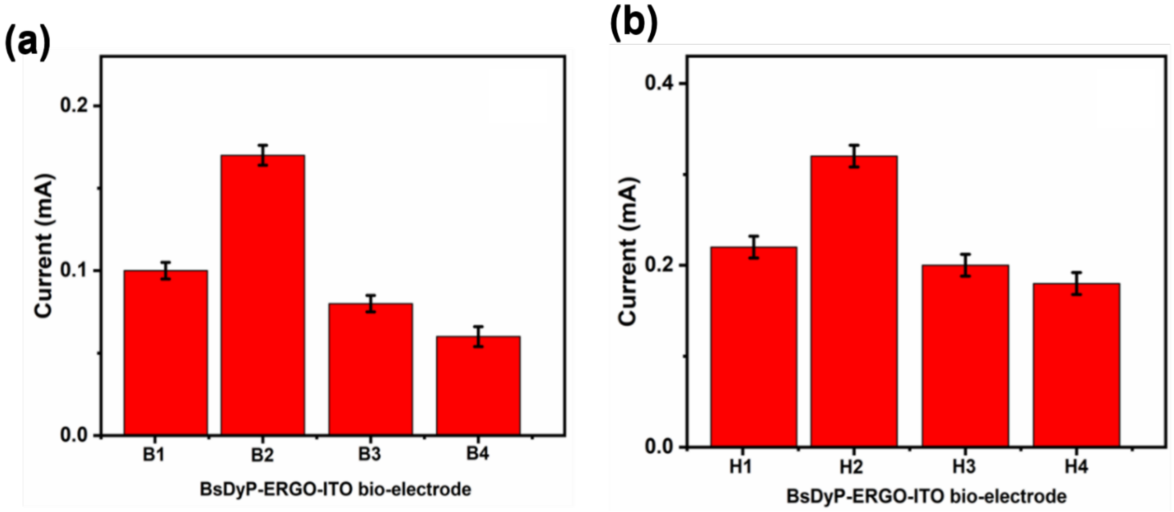

Figure S4. Ipa recorded for the B1, B2, B3, and B4 BsDyP-ERGO/ITO bioelectrodes in presence of identical 5 µM of H_2O_2 solution (100 µL) containing 0.2 M acetate buffer supplemented with 5 mM $[Fe(CN)_6]^{3-/4-}$. DC voltage, ranging from −0.5 V to 1.2 V with γ of 100 mV s^{-1} was applied to the working electrode.

2. Bioinformatics analysis and generation of homology model of BsDyP

2.1 Amino acid sequences alignment between PDB 6KMN[3] and the BsDyP from this study (without poly-histidine purification tag)

```
6KMM_1|Chains      -----------------------------------------------------MEEQIVP 7
BSDYP-HIS6         MSDEQKKPEQIHRRDILKWGAVAGAAVAIGASGLGGLAPLVQTAAKSSNKDEKEDEQVVP 60
                                                                         :**:**

6KMM_1|Chains      FYGKHQAGITTAHQTYVYFAALDVTAKEKSDIITLFRNWTSLTQMLTSGKKMSAEQRNQY 67
BSDYP-HIS6         FYGKHQAGITTAHQTYVYFAALDVTAKEKSDIITLFRNWTVLTQMLTSGKKMPAEQKNQY 120
                   **************************************** *********** ***:***

6KMM_1|Chains      LPPQDTGESADLSPSNLTVTFGFGPSFFEKDGKDRFGLKSKKPKHLAALPAMPNDNLDEK 127
BSDYP-HIS6         LPPQDTGESADLSPSNLTVTFGFGSDFFEKDGKDRFGLKNKKPKHLAALPAMPNDNLDEK 180
                   ************************ .*************.********************

6KMM_1|Chains      QGGGDICIQVCADDEQVAFHALRNLLNQAVGTCEVRFVNKGFLSGGKNGETPRNLFGFKD 187
BSDYP-HIS6         QGGGDICIQVCADDEQVAFHALRNLLNQAIGTCEVRFVNKGFLSGGKNGETPRNLFGFKD 240
                   *****************************:******************************

6KMM_1|Chains      GTGNQSTEDDSLMNSIVWVQSGEPDWMTGGTYMAFRKIKMFLEIWDRSSLKDQEDTFGRR 247
BSDYP-HIS6         GTGNQSTEDDTLMNSIVWIQSGEPDWMTGGTYMAFRKIKMFLEIWDRSSLKDQEDTFGRR 300
                   **********:*******:*****************************************

6KMM_1|Chains      KSSGAPFGQKKETDPVKLNQIPSNSHVSLAKSTGKQILRRAFSYTEGLDPKTGYMDAGLL 307
BSDYP-HIS6         KSSGAPFGQKKETDPVKLNQIPSNSHVSLAKSTGKQILRRAFSYTEGLDPKTGYMDAGLL 360
                   ************************************************************

6KMM_1|Chains      FISFQKNPDNQFIPMLKALSAKDALNEYTQTIGSALYACPGGCKKGEYIAQRLLES     363
BSDYP-HIS6         FISFQKNPDNQFIPMLKALSAKDALNEYTQTIGSALYACPGGCKKGEYIAQRLLES     416
                   ********************************************************
```

Fig. S5. Sequence alignment between DyP from *Bacillus subtilis* (PDB 6KMN)[3] and the BsDyP from this study. The initial amino acid chain is missing in PDB 6KMN comparing with the BsDyP from this study. PDB 6KMN and the BsDyP from this study also differ for further 12 amino acid residues.

Percent Identity Matrix - created by Clustal2.1

1: 6KMM_1|Chains	100.00	96.69
2: BSDYP-HIS6	96.69	100.00

2.2 Generation of homology model of BsDyP

The generation of the homology model was carried out using the YASARA[4] (Version 19.12.14.W.64) homology model building protocol,[5] which involves multi-template structural model generation. Since the linear amino acid sequence of the target protein was the only given input, one template was manually provided namely the X-ray crystal structure of another DyP from *B. subtilis* that was recently crystallized and biochemically characterized (PDB 6KMN).[3] The other possible templates were identified by running 3 PSI-BLAST[6] iterations to extract a position specific scoring matrix (PSSM) from UniRef90,[7] and then searching the PDB for a match with an E-value below the entered homology modelling cut-off of 0.5. A maximum of five templates was allowed, therefore the one manually provided plus additional four selected through the bioinformatics search. To aid alignment correction and loop modelling, a secondary structure prediction for the target sequence had to be obtained. This was achieved by running PSI-BLAST to create a target sequence profile and feeding it to the PSI-Pred[8] secondary structure prediction algorithm. For each of the found templates, models were built. Either a single model per template was generated, when the alignment was certain, or a number of alternative models were generated, when the alignment was ambiguous. A maximum of 50 conformations per loop were explored. A maximum of 10 residues were added to the termini. Finally, YASARA tried to combine the best parts of the

generated models to obtain a hybrid model, with the intention of increasing the accuracy beyond each of the contributors. The quality of the models was evaluated by use of Z-score.[9] A Z-score describes how many standard deviations the model quality is away from the average high-resolution X-ray structure. The overall Z-score for the model was calculated as the weighted averages of the individual Z-scores using the formula:

$$\text{Overall} = 0.145 \times \text{Dihedrals} + 0.390 \times \text{Packing1D} + 0.465 \times \text{Packing3D}$$

The overall score thus captures the correctness of backbone (Ramachandran plot) and side-chain dihedrals as well as packing interactions. Notably, the obtained model was already of high quality; therefore, molecular dynamic refinement simulation was not necessary.

Amino acid sequence of BsDyP:

```
MSDEQKKPEQIHRRDILKWGAVAGAAVAIGASGLGGLAPLVQTAAKSSNKDEKEDEQVVPFYGKHQAGITTAHQT
YVYFAALDVTAKEKSDIITLFRNWTVLTQMLTSGKKMPAEQKNQYLPPQDTGESADLSPSNLTVTFGFGSDFFEK
DGKDRFGLKNKKPKHLAALPAMPNDNLDEKQGGGDICIQVCADDEQVAFHALRNLLNQAIGTCEVRFVNKGFLSG
GKNGETPRNLFGFKDGTGNQSTEDDTLMNSIVWIQSGEPDWMTGGTYMAFRKIKMFLEIWDRSSLKDQEDTFGRR
KSSGAPFGQKKETDPVKLNQIPSNSHVSLAKSTGKQILRRAFSYTEGLDPKTGYMDAGLLFISFQKNPDNQFIPM
LKALSAKDALNEYTQTIGSALYACPGGCKKGEYIAQRLLES
```

Table S1: Homology models of the BsDyP peroxidase used in this study. The full amino acid sequence was explored. The multimeric state and the selected templates are shown. Manually imposed template is shown in bold. The accuracy of the generated model is reported by the use of Z-scores. [9]

**Model**	**Multimeric state**	**Templates**	**Number of models**	**Overall Z-score (Quality)*a***
BsDyP peroxidase	dimer	**6KMN**, 6I7C-B, 4GRC-A, 3O72-A, 2Y4D-A	10	-0.862 (Good)

3. Characterization of ERGO/ITO and BsDyP-ERGO/ITO

3.1 General information

The BsDyP peroxidase immobilized on ERGO-deposited ITO surfaces were characterized for purity and optimized for observing uniform layers of ERGO flakes using various advanced nanotechnology tools including Fourier transform infrared (FTIR) spectroscopy (Gladi ATR, Pike technologies). For the FTIR of GO/ITO, sample was prepared by drop-casting GO solution (1 mg mL^{-1}) onto the ITO glass. We used ITO/glass instead of PET to avoid the noise due to PET peaks in the ERGO/ITO sample. Raman, XRD, CV (Auto lab potentiostat/galvanostat), AFM studies were also performed. For Raman and XRD characterization, samples were also prepared on ITO/glass for the same reason as above. Scanning electron microscopy (SEM) images were acquired with a Zeiss Auriga scanning electron microscope. Before imaging, the samples were sputter-coated with platinum/iridium.

3.2 XRD studies

The structural differences between graphite, GO and ERGO/ITO can clearly be seen by comparing their X-Ray Diffraction (XRD) spectra (Supporting information, Fig. S6). In raw graphite, the crystalline peak is found at 2θ = 26.46° (lattice spacing of 0.34 nm) that is its characteristic (002) diffraction peak. After the oxidation to GO, the peak shifts to a lower angle at 2θ = 10.7° (lattice spacing of 0.81 nm). This increase in interlayer spacing is ascribed to the intercalation of water and the presence of oxygen functionalities (epoxide, carboxyl, hydroxyl) on the carbon basal plan sheet. In ERGO/ITO the diffraction peak at 10.7° disappeared and a new peak appeared at 2θ = 26.7° (lattice spacing of 0.34 nm) as a result of the successful electrochemical reduction.

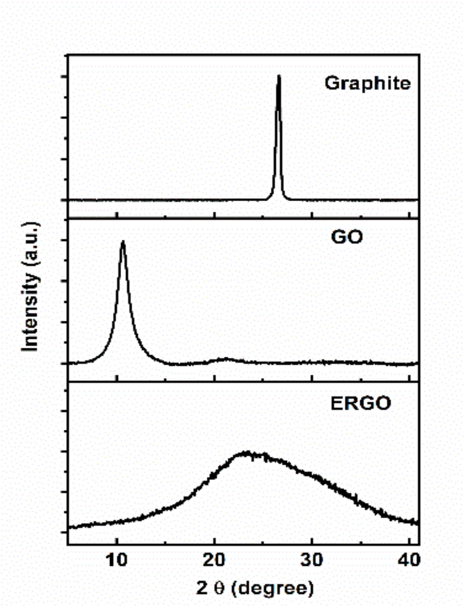

Fig. S6. XRD pattern of graphite, graphene oxide (GO) and electrochemically reduced graphene oxide (ERGO).

3.3 Surface analysis

Table S2. Comparison between surface roughness and root mean square roughness of the GO, ERGO/ITO and BsDyP-ERGO/ITO surface.

**Electrode**	**Surface roughness (Ra) [nm]**	**Root mean square roughness (Rq) [nm]**
GO	19	18
ERGO/ITO	4.2	3.2
BsDyP-ERGO/ITO	90	60

3.4 Electrochemical characterization

Table S3. Electrochemical results of the electrodes.

**Electrode**	**Potential difference (ΔE) [V]**	**Anodic-cathode peak current ratio (I_{pa}/I_{pc})**
ITO/PET	0.25	0.83
ERGO/ITO/PET	0.24	0.86
BsDyP-ERGO/ITO/PET	0.28	0.88

The CV scans were recorded in 0.2 M acetate buffer (pH = 4) containing 5 mM $[Fe(CN)_6]^{3-/4-}$

Table S4. Electrochemical response of BsDyP-ERGO/ITO/PET biosensor at H_2O_2 concentration ranging from 0.05 µM to 320 µM. The CV scans were recorded in 0.2 M acetate buffer containing 5 mM $[Fe(CN)_6]^{3-/4-}$ at a scan rate of 50 mV s^{-1}.

**Concentration [µM]**	**Current intensity Ipa [A]**	**Standard deviation**
0.05	1.60×10^{-4}	9.50×10^{-6}
0.1	1.80×10^{-4}	1.15×10^{-5}
1	1.84×10^{-4}	1.15×10^{-5}
10	1.90×10^{-4}	1.45×10^{-5}
40	2.20×10^{-4}	1.60×10^{-5}
80	2.60E-04	1.80×10^{-5}
120	3.00×10^{-4}	1.15×10^{-5}
160	3.20×10^{-4}	1.45×10^{-5}
200	3.60×10^{-4}	1.60×10^{-5}
240	3.90×10^{-4}	1.80×10^{-5}
280	4.20×10^{-4}	1.15×10^{-5}
320	4.23×10^{-4}	1.45×10^{-5}

a) The linear regression within the range of linearity (0.05–280 µM) afforded the following equation:

$I_{pa} = 4.5 \times 10^{-4} + (8.8 \times 10^{-7})\, C_t$

where I_{pa} is the current intensity in Ampere and C_t is the concentration of H_2O_2 (µM).

The R^2 coefficient of the linear regression was 0.985.

b) The determination of the limit of detection was performed via the "$3Sb/m$" criteria

$$LOD = \frac{3 \times S_b}{m} = \frac{3 \ \times 9.5 \times 10^{-6}}{8.8 \times 10^{-7}} = 32\ nM$$

3.5 Calculation of the percentage of interference

The percentage (%) of current change (I_{pa} values) with various substances (100 µL of interferants, 200 µM concentration) was determined in acetate buffer (pH 4, 0.2 M) containing 100 µL of a 200 µM H_2O_2 solution (i.e., a 1:1 ratio of H_2O_2 to interferant). The percentage interference (% I_{int}) is calculated for each interferant using the following equation:

$$\%I_{int} = \frac{I_{int+H_2O_2} - I_{H_2O_2}}{I_{H_2O_2}} \times 100$$

wherein $I_{H_2O_2}$ is the anodic peak current after the addition of 100 µL of a 200 µM H_2O_2 solution, and $I_{int+H_2O_2}$ is the current recorded after the addition of a 1:1 mixture of H_2O_2 and interferant.

Table S5. Comparison of present biosensor with the existing ones, all working in presence of a mediator.

Electrode	Linear range	Detection limit	Sensitivity	Stability	References
HRP/MB/GE	10–560 μM	3 μM	–	5 days	[10]
TH/HRP/SAME/Au	40–100 μM	40 μM	2.5×10^{-3} A M^{-1} cm^{-2}	–	[11]
HRP/nano-Au/CCE/GC	12.2 μM to 1.1 mM	6.1 μM	0.29 A M^{-1} cm^{-2}	2–5 weeks	[12]
GNP-TNT/HIL/HRP/GC	15–750 μM	2.2 μM	0.228 A M^{-1} cm^{-2}	2 weeks	[13]
HRP/PEGDGE/[Os(dmp)PVI]$^{+/2+}$/G	15–500 μM	0.3 μM	0.297 A M^{-1} cm^{-2}	4 weeks	[14]
BsDyP-ERGO/ITO	0.05–280 μM	32 nM	3.5 A M^{-1} cm^{-2}	9 weeks	This work

GE: Graphite Electrode; MB: Methylene Blue; HRP: Horseradish Peroxidase; MWCNT-NF: multiwalled carbon nanotubes-Nafion; CAT: catalase; PEI: polyethylenimine; GCE: glassy carbon electrode; nano-Au: gold nanoparticles; CCE: carbon ceramic electrode; GNP-TNT: gold nanoparticles- dotted TiO2 nanotubes; HIL: hydrophobic ionic liquid (1-decyl-3-methylimidazolium tetrafluoroborate); PEGDGE: poly(ethyleneglycol) diglycidyl ether; [Os(dmp)PVI]$^{+/2}$: [Osmium(4,4′-dimethyl-2,2′-bipyridine)$_2$ poly(N-vinylimidazole)$_{10}$Cl]$^{+2/+}$.

4. References

[1] K. Min, G. Gong, H. M. Woo, Y. Kim, Y. Um, *Sci. Rep.* **2015**, *5*, 8245.

[2] a) W. S. Hummers, R. E. Offeman, *J. Am. Chem. Soc.* **1958**, *80*, 1339-1339; b) S. K. Bhardwaj, P. Yadav, S. Ghosh, T. Basu, A. K. Mahapatro, *ACS Appl. Mater. Interfaces* **2016**, *8*, 24350-24360.

[3] P. Dhankhar, V. Dalal, J. K. Mahto, B. R. Gurjar, S. Tomar, A. K. Sharma, P. Kumar, *Arch. Biochem. Biophys.* **2020**, *693*, 108590.

[4] E. Krieger, G. Koraimann, G. Vriend, *Proteins: Struct. Funct. Genet.* **2002**, *47*, 393-402.

[5] H. Venselaar, R. P. Joosten, B. Vroling, C. A. Baakman, M. L. Hekkelman, E. Krieger, G. Vriend, *Eur. Biophys. J.* **2010**, *39*, 551-563.

[6] S. Altschul, *Nucleic Acids Res.* **1997**, *25*, 3389-3402.

[7] B. E. Suzek, H. Huang, P. McGarvey, R. Mazumder, C. H. Wu, *Bioinformatics* **2007**, *23*, 1282-1288.

[8] D. T. Jones, *J. Mol. Biol.* **1999**, *292*, 195-202.

[9] a) R. A. Laskowski, M. W. MacArthur, D. S. Moss, J. M. Thornton, *J. Appl. Crystallogr.* **1993**, *26*, 283-291; b) R. W. Hooft, G. Vriend, C. Sander, E. E. Abola, *Nature* **1996**, *381*, 272.

[10] X.-Q. Lin, J. Chen, Z.-H. Chen, *Electroanalysis* **2000**, *12*, 306-310.

[11] C. Ruan, F. Yang, C. Lei, J. Deng, *Anal. Chem.* **1998**, *70*, 1721-1725.

[12] C. X. Lei, S. Q. Hu, N. Gao, G. L. Shen, R. Q. Yu, *Bioelectrochemistry* **2004**, *65*, 33-39.

[13] X. Liu, H. Feng, J. Zhang, R. Zhao, X. Liu, D. K. Wong, *Biosens. Bioelectron.* **2012**, *32*, 188-194.

[14] P. Bollella, L. Medici, M. Tessema, A. A. Poloznikov, D. M. Hushpulian, V. I. Tishkov, R. Andreu, D. Leech, N. Megersa, M. Marcaccio, L. Gorton, R. Antiochia, *Solid State Ion.* **2018**, *314*, 178-186.
